# Illumina Sequencing in Conjunction with Propidium Monoazide to Identify Live Bacteria After Antiseptic Treatment in a Complex Oral Biofilm: A Study Using an Ex Vivo Supragingival Biofilm Model

**DOI:** 10.3390/antibiotics13111000

**Published:** 2024-10-23

**Authors:** María C. Sánchez, Paola Hernández, Ángela Velapatiño, Eber Cuba, María J. Ciudad, Luis Collado

**Affiliations:** 1 Department of Medicine, Faculty of Medicine, University Complutense, 28040 Madrid, Spain; mariasan@ucm.es (M.C.S.); leidypah@ucm.es (P.H.); angvelap@ucm.es (Á.V.); ebercuba@ucm.es (E.C.); lcollado@ucm.es (L.C.); 2 GINTRAMIS Research Group (Translational Research Group on Microbiota and Health), Faculty of Medicine, University Complutense, 28040 Madrid, Spain

**Keywords:** oral biofilm, cariogenic biofilm, Illumina 16S rRNA sequencing, propidium monoazide, chlorhexidine, cetylpyridinium chloride

## Abstract

**Background/Objectives**: The evaluation of the efficacy of antibacterial treatments in complex oral ecosystems is limited by the inability to differentiate live from dead bacteria using omic techniques. The objective of this study was therefore to assess the ability of the combination of the 16S rRNA Illumina sequencing methodology and the action of propidium monoazide (PMA) to study viable bacterial profiles in oral biofilms after exposure to an antiseptic compound. **Methods**: Cariogenic supragingival biofilms were developed in an ex vivo model for 96 h, using saliva from healthy volunteers. The biofilms were treated with 0.12% chlorhexidine (CHX) combined with 0.05% cetylpyridinium chloride (CPC), for 60 s, using phosphate buffered saline as a control. After exposure, each biofilm was treated or not with PMA to then extract the bacterial DNA, quantify it by Qubit, quantify the bacterial population using qPCR, and perform the metataxonomic study of the samples using Illumina 16S rRNA sequencing. **Results**: A significantly lower DNA concentration in the PMA-treated biofilms (*p* < 0.05 compared with those not exposed to PMA) was observed. The viable bacterial count obtained by qPCR differed significantly from the total bacterial count in the biofilm samples exposed to the antiseptic (*p* < 0.05). The viable microbiome differed significantly from the total bacterial profile of the samples treated with CHX/CPC after exposure to PMA (*p* < 0.05 at the α- and β-diversity levels). **Conclusions**: The combination of Illumina 16S rRNA sequencing and PMA helps solve the inability to evaluate the efficacy of antibacterial treatments in the bacterial profile of complex ecosystems such as oral biofilms.

## 1. Introduction

Oral biofilms and their associated diseases represent a high risk for public health [1,2,3]. With the progress in biological and molecular diagnostic techniques, evidence is accumulating that, with regard to health promotion and disease, we are still far from understanding the complex host–oral microbiome interaction, as well as the interaction between different microorganisms that shape oral biofilms and the interaction between these and their environment.

The pathogenic potential of these polymicrobial communities, mainly bacterial, was initially associated with the presence of specific pathogens. However, evidence is emerging that the sole presence of certain key bacterial species is insufficient for triggering the disease but that a change in their relative abundance, caused by a wide range of factors, could induce a change in the community’s microbial structure, causing microbiota to change from symbiotic to dysbiotic, thereby instigating the disease [4]. Thus, for example, the presence of *Porphyromonas gingivalis* in the human periodontium does not always lead to the conversion of a symbiotic microbiome to a dysbiotic one. This periodontal pathogen can be detected at low levels in the normal microbiota of healthy individuals; in individuals with periodontal disease, however, significant changes are detected in the global microbial composition of the dental plaque, revealing an increase in the abundance of *P. gingivalis* associated with a reduction in the abundance of beneficial bacteria [3]. There is also evidence that dysbiosis of the commensal bacteria of these ecosystems and the consequent change in their relative abundance could act as an etiological factor in the onset and development of the various medical conditions associated with bacterial action in the oral cavity [1,2,3,4,5,6]. Thus, at the supragingival level, for example, infection by *Streptococcus mutans* is considered associated with the caries process [7,8]. However, other species of non-mutans streptococci and their change in abundance in the supragingival plaque, including *Streptococcus sobrinus*, *Streptococcus salivarius*, and *Streptococcus parasanguinis*, as well as several species of the genera *Lactobacillus*, *Actinomyces*, *Bifidobacterium*, *Veillonella*, *Propionibacterium*, and *Atopobia*, all of them a priori commensal species, start to emerge as potentially responsible for dental caries disease in its different stages [9,10,11,12,13,14].

The obvious association between the dysbiosis of dental plaque and the onset and development of oral diseases, coupled with the need for managing these bacterial communities in terms of prevention and treatment, establishes the need to work with techniques that enable the observation of the dynamics and overall response of the community and not focus solely on one or several bacterial species [2,3,6]. The methods based on bacterial cultures and microscopy observation, which help obtain a complete view of a sample’s microbiota and identify pathogenic bacteria, methods that have been considered for decades as the reference techniques, have the drawbacks of being labor-intensive, time-intensive and, in many cases, unable to detect difficult-to-cultivate species and demanding or slow-growing microorganisms, which are highly abundant in the oral cavity [15,16]. In contrast, the development of culture-independent molecular techniques helps clinicians work with genetic material obtained directly from environmental samples, without subjecting the microorganisms to growth in the laboratory, which has helped advance the understanding of population dynamics, providing a more complete view of the changes that occur in these microbial communities [17,18].

At the oral cavity level, molecular techniques based on nucleic acids such as DNA–DNA hybridization, DNA microarrays, the various polymerase chain reaction (PCR) modalities, and next-generation sequencing, along with other genomic, metagenomic, phylogenomic, pangenomic, and transcriptomic techniques, as well as techniques based on mass spectrometry, metabolomics, proteomics, and lipidomics, among others, have expanded the understanding of the role of both culturable and non-culturable bacteria in the onset and development of oral diseases and the understanding of the associated bacterial dynamics [1,3,18]. Nowadays, advances in sequencing technology position Illumina as the most widely used technique in research, compared to third-generation techniques, which are still expanding and have high costs (especially long-read technologies such as nanopore sequencing (Oxford Nanopore, Oxford, UK), single-molecule real-time sequencing (SMRT), and LoopSeq (Element Biosciences, San Diego, CA, USA)). This technique can massively generate millions of DNA fragments simultaneously in a single sequencing process, significantly increasing throughput at a lower cost, offering significant advantages over conventional systems. This allows many more researchers to conduct larger-scale 16S gene sequencing projects, metagenomics, whole genome sequencing, and RNA sequencing [1,3,18].

Despite the benefits of avoiding microbial cultures and working with the total bacterial genetic material present in a sample, the results of these techniques can be compromised. This is the case with the culture-independent techniques based on DNA amplification, which overestimate the diversity of species, both in the richness of species and in their relative abundance, by not discriminating between the DNA of live microbial cells (latent cells, as well as metabolically active cells in growth or non-growth) and dead cells [19]. This fact hinders the evaluation of the efficacy of immediate-acting active compounds employed in therapies or in preventive actions given that the DNA of dead bacterial cells can persist in the environment and thereby result in false positives [19].

To address this deficiency, culture-independent methods have recently been combined with a DNA intercalating agent, propidium monoazide (PMA). PMA is a cell membrane-impermeable and photo-reactive dye that binds to DNA with high affinity. Upon photolysis with visible light, PMA dye becomes covalently attached to DNA. This modified DNA cannot be amplified by PCR. Thus, PMA selectively prevents DNA amplification of dead cells as well as that of free double-stranded DNA in the environment [20,21,22]. There are numerous studies to date that have optimized the use of PMA combined with qPCR with regard to variables such as PMA concentration, incubation duration and temperature, and the environmental characteristics under study, including the oral cavity [23,24,25,26,27,28,29,30]. However, there have been very few studies that have explored the ability to combine methodologies based on high-throughput sequencing and PMA for detecting live cells in human and environmental samples, some as complex as the oral cavity and the biofilms it hosts [21,22,31,32,33,34,35,36,37].

Therefore, this study’s objective was to assess the applicability of combining the Illumina 16S rRNA high-throughput sequencing methodology with PMA as a useful tool for evaluating the efficacy of an antiseptic treatment on the richness and relative abundance of bacterial species that make up the oral biofilm.

## 2. Results

### 2.1. The PMA-Exposed Oral Biofilms Contain a Significantly Lower Concentration of DNA

Table 1 lists the concentrations of bacterial DNA, expressed as mean and standard deviation (SD) in ng/µL, from the biofilms treated for 60 s with PBS (control biofilms) or with the CHX/CPC antiseptic (treated biofilms) and then exposed or not to PMA (final concentration 100 µM).

The results confirmed that the control biofilms treated with PBS, once exposed to PMA, showed significantly less bacterial DNA than the same non-exposed samples (5.9 (2.4) and 14.1 (4.4) ng/µL, respectively; 95% CI −15.4–−0.90; *p* = 0.027) (Table 1). Similarly, the biofilms treated with the CHX/CPC antiseptic presented a significant bacterial DNA concentration when exposed to PMA compared with the same non-exposed samples (0.16 (0.1) and 3.1 (1.7) ng/µL, respectively; 95% CI −5.9–−0.006; *p* = 0.050) (Table 1). In contrast, we confirmed that the biofilms treated with CHX/CPC but not exposed to PMA showed no significant differences in bacterial DNA concentration compared with the control biofilms exposed to PMA (3.1 (1.7) and 5.9 (2.4) ng/µL, respectively; 95% CI −6.95–−1.27; *p* = 0.244) (Table 1).

The effectiveness of the antiseptic in the biofilms was revealed in terms of DNA concentration with the significant differences when comparing the DNA concentration of the biofilms treated with CHX/CPC compared with PBS treatment, both exposed to PMA (0.16 (0.1) and 5.9 (2.4) ng/µL, respectively; *p* = 0.013) (Table 1).

### 2.2. The Viable Bacterial Count Obtained by qPCR Differed from the Total Bacterial Count (Live and Dead Bacteria) in the Biofilm Samples Exposed to the Antiseptic

Table 1 shows the quantification of the bacteria, expressed as mean (SD) CFU/mL, from the biofilms treated for 60 s with PBS (control biofilms) or the CHX/CPC antiseptic (treated biofilms), then exposed or not to PMA (final concentration 100 µM).

First, we were able to verify that the biofilms treated with PBS and then exposed to PMA showed a bacterial count (corresponding to the portion of viable bacteria in the biofilms) that was lower but not significantly lower than those not exposed to PMA, which reflects the total bacterial count (viable and non-viable) (1.0 × 10^9^ (9.7 × 10^8^) and 1.4 × 10^9^ (6.1 × 10^8^) CFU/mL, respectively; *p* = 1.000) (Table 1), which suggests the presence of a non-significant portion of dead bacteria in the oral biofilms at 96 h of incubation.

Moreover, when checking the effect of the antiseptic on bacterial viability, the results suggest that the qPCR technique is unable to discern between the DNA from viable and non-viable bacteria and can overestimate the bacterial count. The qPCR bacterial count was non-significantly reduced (*p* = 1.000) in the biofilms treated with CHX/CPC (9.0 × 10^8^ (6.4 × 10^8^) CFU/mL) compared with the control (1.40 × 10^9^ (6.1 × 10^8^) CFU/mL) (Table 1). However, when exposing the biofilms to PMA, these results changed significantly. After the treatment with the CHX/CPC antiseptic and subsequent exposure to PMA, the bacterial count (5.0 × 10^6^ (1.1 × 10^7^)) was significantly lowered compared with the other samples, that is, compared with the control biofilms exposed to PBS, with and without PMA (1.0 × 10^9^ (9.7 × 10^8^) and 1.4 × 10^9^ (6.1 × 10^8^) CFU/mL, respectively; *p* = 0.048 and *p* = 0.002, respectively), and with the biofilms treated with CHX/CPC but without exposure to PMA (9.0 × 10^8^ (6.4 × 10^8^) CFU/mL; *p* = 0.003) (Table 1).

### 2.3. The Viable Microbiome Differs from the Total Bacterial Profile in the Oral Biofilm Samples After Exposure to an Active Antiseptic Ingredient

**Figure 1:**
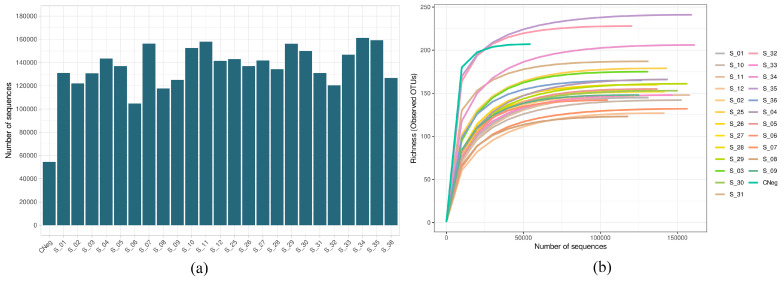
(**a**) Number of sequences after quality filtering the cariogenic supragingival biofilm samples developed in an ex vivo model for 96 h from the saliva of healthy volunteers. The biofilms were treated with 0.12% chlorhexidine combined with 0.05% cetylpyridinium chloride (CHX/CPC), for 60 s, using phosphate buffered saline (PBS) as a control. After exposure, each biofilm was treated or not with propidium monoazide (PMA). All samples were sequenced successfully, with the number of reads exceeding 1,000,000 for all samples. (**b**) Rarefaction curves obtained at the amplicon sequence variant level of the above-mentioned samples. All curves were saturated, which confirmed that the sequencing depth was appropriate for detecting all existing diversity in the samples.

Figure 1a shows the number of sequences obtained in the sequencing process after quality filtering in the biofilms obtained by the Illumina Miseq technique, from the biofilms treated for 60 s with PBS (control biofilms) or with antiseptic (treated biofilms), subsequently exposed or not to PMA (final concentration 100 µM). The number was greater than 100,000 in all samples except for the negative control. The mean value of sequences by sample was 135,292.3 ± 4455.7 (minimum value, 54,523; maximum value, 161,167) (Figure 1a). Moreover, all rarefaction curves were saturated, indicating that the sequencing depth employed was sufficient for detecting practically all of the existing diversity (Figure 1b).

**Figure 2:**
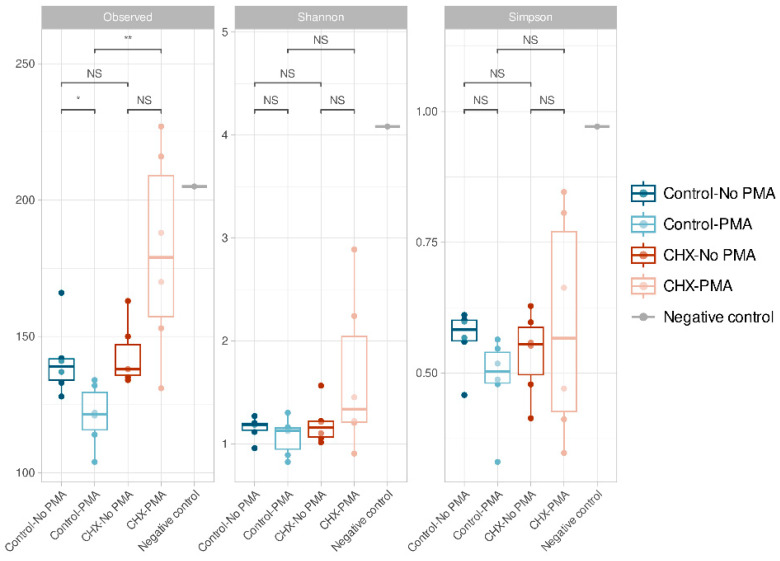
Amplicon sequence variant α-diversity in the various groups of cariogenic supragingival biofilms developed in an ex vivo model for 96 h, from the saliva of healthy volunteers. The biofilms were treated with 0.12% chlorhexidine combined with 0.05% cetylpyridinium chloride (CHX/CPC), for 60 s, using phosphate buffered saline (PBS) as a control. After exposure, each biofilm was treated or not with propidium monoazide (PMA). The results of the Wilcoxon test performed between the various groups are shown with the following coding: NS (not significant; *p* > 0.05); * (*p* < 0.05); ** (*p* < 0.01). The oral biofilm samples presented low diversity and were, in general terms, similar.

The α-diversity, which is analyzed using three indices (richness, Shannon’s diversity, and Simpson’s diversity), demonstrated that all samples presented low α-diversity values and, in general, were very similar to each other (Figure 2). We confirmed that the control PBS-treated biofilms and those treated with CHX/CPC presented no significant differences in ASV richness (*p* > 0.05). In contrast, when comparing the group of CHX/CPC-treated samples exposed to PMA against the PBS-treated control group also exposed to PMA, there were significant differences in the number of ASVs observed (*p <* 0.01 in the Wilcoxon test) (Figure 2). In this regard, there were a number of samples treated with CHX/CPC and PMA that presented greater ASV richness (Figure 2).

In the group of PBS-treated control samples, there were also significant differences in ASV richness when comparing the samples exposed and those not exposed to PMA, the latter showing an increased number of observed ASVs (*p* < 0.05 in the Wilcoxon test). There were no significant differences in the rest of the comparisons or when comparing the Shannon and Simpson indices.

**Figure 3:**
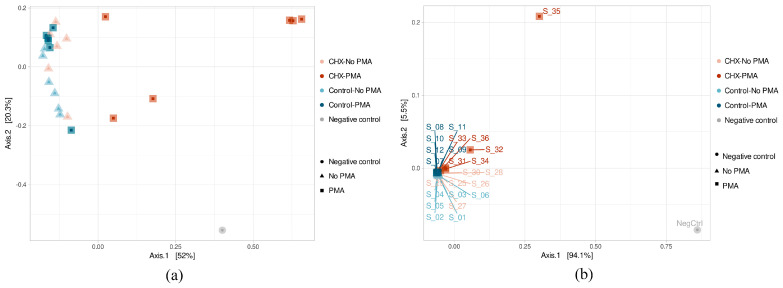
(**a**,**b**) Amplicon sequence variant β-diversity of the samples of cariogenic supragingival biofilms and negative control, developed in an ex vivo model for 96 h from the saliva of healthy volunteers. The biofilms were treated with 0.12% chlorhexidine combined with 0.05% cetylpyridinium chloride (CHX/CPC) for 60 s, using phosphate buffered saline (PBS) as a control. After exposure, each biofilm was treated or not with propidium monoazide (PMA).

The β-diversity study was performed using the principal component analysis (PCoA) after calculating a dissimilarity matrix between samples with the Bray–Curtis method. The results of the ASV β-diversity analysis showed, above all, that three samples belonging to the group treated with CHX/CPC and exposed to PMA (S_31, S_34, and S_36) were considerably differentiated in their microbial composition (Figure 3a). The rest of the samples were grouped in the same part of the X axis, thereby showing greater homogeneity in their composition. Although they were not clearly grouped according to the type of treatment applied, the samples belonging to the same group did appear to stay close to each other, as was the case for the PBS-treated samples unexposed to PMA and also those exposed, as well as the samples treated with CHX/CPC but not PMA. The PERMANOVA test confirmed that both the type of treatment (PBS or CHX/CPC) and exposure to PMA (applied or not applied) were variables that significantly affected the samples’ microbial composition (*p* < 0.05 in all cases).

The β-diversity at the genus level showed that two samples treated with CHX/CPC and PMA (S_32 and S_35) differed the most from the others, coinciding with the lower bacterial concentration from the biofilm. The remaining samples appeared highly grouped with each other, except the remaining samples treated with CHX/CPC and PMA (S_31, S_34, and S_36), which were slightly displaced within this grouping (Figure 3b). Therefore, this group formed by samples treated with CHX and PMA differed the most from the rest, although it was also not homogeneous in its composition, as can be seen from the obvious non-grouping of its samples in the PCoA (Figure 3b).

As regards the bacterial profiles and at the phylum level, there was a noteworthy predominance of *Firmicutes* in all samples. Thus, in the PBS-treated group with or without exposure to PMA and in the CHX/CPC-treated group without exposure to PMA, the mean relative abundance of this phylum was close to 99%, while in the CHX/CPC-treated group with PMA, the abundance decreased significantly up to 92%. In terms of the differential abundance analysis among the phyla, the control biofilms had no observable phylum differentially present among the samples exposed to PMA and those not exposed. In those treated with CHX/CPC, the phylum *Actinobacteriota* was statistically more present in the samples treated with PMA (*p* = 0.005). This phylum was also more abundant in the samples treated with CHX/CPC and PMA than in the samples treated with PBS and exposed to PMA (*p* = 0.001) along with the phyla *Proteobacteria* (*p* = 0.004) and *Bacteroidota* (*p* = 0.01).

**Figure 4:**
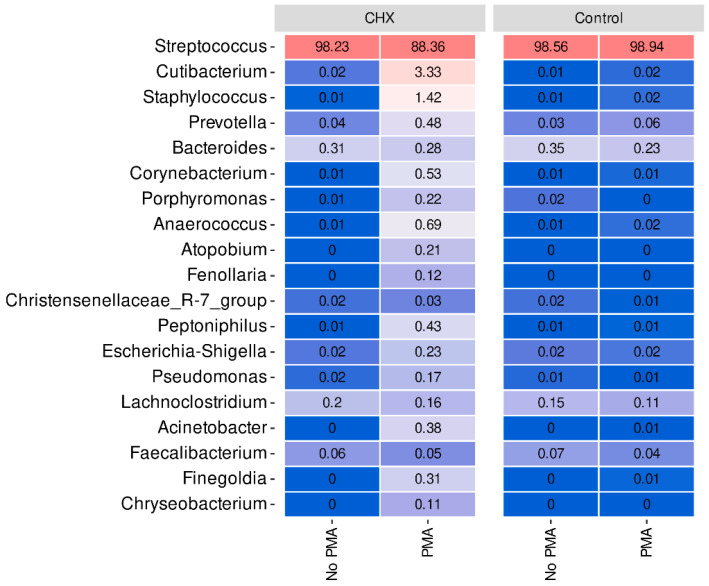
Taxonomic distribution at the genus level of the samples of cariogenic supragingival biofilms developed in an ex vivo model for 96 h, from the saliva of healthy volunteers. The biofilms were treated with 0.12% chlorhexidine combined with 0.05% cetylpyridinium chloride (CHX/CPC), for 60 s, using phosphate buffered saline (PBS) as a control. After exposure, each biofilm was treated or not with propidium monoazide (PMA). *Streptococcus*, the genus typically predominant in the human oral cavity, was clearly dominant in all samples.

At the genus level, we verified that *Streptococcus*, a genus belonging to the phylum *Firmicutes*, predominated very clearly in all samples of the dysbiotic biofilms generated ex vivo (Figure 4). In fact, this genus represents more than 98% of the mean relative abundance in the control group treated or not with PMA and in the CHX/CPC-treated group without PMA treatment, reducing to 88% on average in the CHX/CPC-treated group exposed to PMA (Figure 4). In the CHX/CPC-treated group with PMA, we observed that, coinciding with the PCoA analysis at the genus level, two samples (S_32 and S_35) presented other genera in their composition with notable relative abundances, as was the case for *Cutibacterium*, *Staphylococcus*, *Prevotella*, and *Corynebacterium*.

The differential abundance analysis of genera among the groups revealed that, when comparing the control biofilms exposed or not to PMA, the genera *Haemophilus*, *Porphyromonas*, and *Gemella* reduced their presence in the samples exposed to the action of PMA (*p* < 0.001 in all cases), while only one genus, CAG-352, increased its relative abundance (*p* = 0.002). Similarly, in the CHX/CPC-treated samples, the comparison between the samples exposed or not to PMA revealed various genera that were more abundant in the samples exposed to PMA, highlighting *Pseudomonas*, *Prevotella*, and *Corynebacterium*, and a single less present genus, Acinetobacter (*p* < 0.05 in all cases). Lastly, when analyzing the effect of the antiseptic compared with the control, both without exposure to PMA, we observed that two genera reduced their presence in the biofilms after treatment with the antiseptic: *Desulfovibrio* and *Peptostreptococcus* (*p* < 0.001 in both cases) (Figure 4).

**Figure 5:**
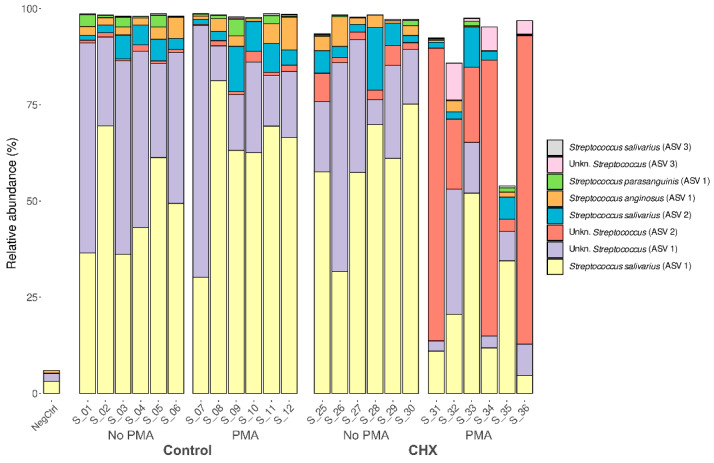
Taxonomic distribution in the amplicon sequence variants of predominant streptococci (mean relative abundance >0.1% and present in at least half of the samples) of the cariogenic supragingival biofilms developed in an ex vivo model for 96 h, from the saliva of healthy volunteers. The biofilms were treated with 0.12% chlorhexidine combined with 0.05% cetylpyridinium chloride (CHX/CPC), for 60 s, using phosphate buffered saline (PBS) as a control. After exposure, each biofilm was treated or not with propidium monoazide (PMA). We verified that it was not a single strain of *Streptococcus* that colonized the biofilms but rather that several comprised the microbiota.

Given the predominance of the *Streptococcus* genus and the involvement of various species in the development of oral cariogenic disease, we studied whether this was a single clone of the *Streptococcus* genus that dominated the biofilm samples or whether it was several. To this end, we analyzed the relative abundance of each ASV detected. Up to 134 distinct ASVs were detected belonging to this genus, present in at least one of the analyzed samples. Of these, eight showed a mean relative abundance greater than 0.1% in the samples and were present in at least half of the oral biofilm samples (Figure 5). This finding, along with the already described results, indicates that the cariogenic biofilm developed in the in vitro model was dominated almost exclusively by different strains of this genus. The most abundant ASV was the species *S. salivarius*, representing 30–70% of the relative abundance in the control samples exposed or not to PMA and in the samples treated with CHX/CPC but unexposed to PMA. In contrast, the presence of species *S. salivarius* was lower in most of the samples treated with CHX/CPC and exposed to PMA. Two other species of the genus found among the most abundant in the biofilms, although with much lower abundance than *S. salivarius*, were *S. anginosus* and *S. parasanguinis* (Figure 5).

There were significant differences when performing the differential abundance analysis of ASVs among the various groups. This was the case for *S. salivarius*, which in the samples with CHX/CPC and PMA was significantly less present than in the other samples (*p* < 0.01 in all cases). The opposite tendency was observed with *Streptococcus* spp., of unknown species, which were clearly more abundant in the samples treated with CHX/CPC and PMA than in the rest of the samples (*p* < 0.001 in all cases). Additionally, when comparing the set of samples untreated with CHX/CPC against the treated ones, we observed that *S. parasanguinis* reduced its abundance in the treated samples (*p* = 0.035).

## 3. Discussion

The study showed that the combination of Illumina 16S rRNA sequencing and PMA helps solve the inability to evaluate the efficacy of antibacterial treatments on the viable microbiome of complex ecosystems such as oral biofilms. We were also able to verify the efficacy of the combination of the active ingredients CHX and CPC (12% and 0.05%, respectively) in managing dysbiotic supragingival biofilms, significantly reducing not only the viable bacteria load but also helping to balance its microbiota towards a condition compatible with healthy dental plaque (*p* = 0.002).

The onset and development of oral disease caused by bacteria is facilitated by a synergistic or cooperative process between species within oral biofilms, with changes in their relative abundance, which play a leading role in defining the pathogenicity of this community [3,38,39]. This impels the need for implementing techniques that help study the response of the sum of bacteria that compose the community and not of a single or several selected species. In this scenario, omic techniques are essential and include those based on culture-independent bacterial DNA sequencing, which allow for rapid and deep characterization of the complete microbial community that inhabits various environments, among them the human body [40,41]. The application of these techniques is revealing a close and mutual interaction between the microorganisms themselves and between the microorganisms and their hosts [41,42,43,44,45,46] and solves the drawbacks resulting from culture-dependent methods [15,16]. However, as determined in the present study, the culture-independent methodologies are not without drawbacks and can introduce inherent biases.

Firstly, we detected a bias resulting from bacterial contamination, which was introduced possibly during the processing and/or analysis of the samples. The increased sensitivity of analysis techniques has made it possible to detect even traces of DNA in clinical and environmental samples. Since sequencing protocols require multiple steps of handling and manipulation of samples in the laboratory, it is not surprising that exogenous DNA may be detected, potentially even in greater quantities than the DNA being studied. While this type of contamination in the laboratory is usually highly controlled through the implementation of routine aseptic procedures, cleaning protocols, and strict methodologies to minimize risks, it is well-documented that appreciable and significant levels of contamination can be detected on laboratory work surfaces, on non-disposable materials, and in analysis equipment. The bacterial diversity of the environment will be reflected in the contaminating DNA traces [47,48]. In this regard, the negative control, even while presenting a low DNA load and fewer than 100,000 sequences in the sequencing process (Figure 1), was the sample with the highest Shannon and Simpson indices and was among those with the highest number of distinct ASVs (Figure 2). We hypothesize that the extraction kit itself or the handling of the samples throughout the study phases might have been sources of contamination, as has been reported by other authors [47]. The β-diversity analysis revealed, however, the major differences in terms of existing microbial communities between the negative control and the biofilms (Figure 3), which indicated the scarce influence of this residual contamination in the biofilms. We verified that the negative control was located far from the other samples, although it appeared to present a certain tendency to approach on the X axis of the PCoA (which explained most of the percentage variation) the three samples of the group treated with CHX/CPC and PMA (Figure 3). These samples presented a low DNA concentration after the extraction (Table 1) and were therefore more susceptible to contamination with the bacterial DNA possibly present in the extraction kits or in the environment during the processing of the samples.

Secondly, another of the biases detected in the culture-independent techniques was the inability to exclude DNA from non-viable cells, which is consistent with reports from other authors in different environments [49,50,51,52], which would entail an inaccurate characterization of its composition and an overestimation of its richness in bacterial species or in its relative abundance, as we were able to determine in this study (Table 1, Figure 2, Figure 3, Figure 4 and Figure 5). Moreover, we were able to confirm that the control biofilms hosted a total bacteria count of 1.4 × 10^9^ (6.1 × 10^8^) CFU/mL (Table 1). By exposing the same samples to the action of PMA, we observed that these communities hosted a non-significant percentage of dead bacteria (approximately 28.6% compared with the total count; *p* = 1.000), which allows us to clearly observe the antiseptic’s effect on the relative abundance of the various species present (Table 1). These results agree with those of other studies that have indicated that the number of viable bacteria is reduced with the aging of the biofilms. A young biofilm hosts approximately 80% of viable bacterial cells, as in our case with approximately 71.4% bacterial viability, while a mature biofilm has approximately 50% [53]. The inability to discern was even clearer after applying the antiseptic treatment to the biofilms. We confirmed that, in the absence of PMA and after the antiseptic treatment, there were no significant qPCR differences between the total number of bacteria of the PBS-exposed biofilms (control biofilms) and those treated with CHX/CPC (*p* = 1.000) (Table 1). However, when applying PMA to both types of biofilms, we verified through qPCR that there was a significant reduction in the number of viable bacteria resulting from the therapeutic intervention (*p* = 0.048) (Table 1). The inability of qPCR to differentiate between viable DNA and that from unviable cells has already been reported by other authors in various environments, including the oral cavity, in various scenarios, including after exposure to antiseptic agents [20,21,22,23,24,25,26,27,28,29,30].

However, there are only a few and very recent studies, such as the one we are presenting, that aim to demonstrate how the combination of various metataxonomic techniques with PMA can show the significant differences that affect the bacterial composition of a community (in its α- and β-diversity) and that cannot be shown by other methods [32,36,37,52,54,55,56] (Figure 2, Figure 3, Figure 4 and Figure 5). Despite the advances achieved, there is no proof in the oral cavity of the efficiency and sensitivity of the combination of omic techniques and PMA when analyzing the effect of various therapeutic or preventive interventions. A recent report on the use of PMA combined with the shotgun metagenomic technique in saliva samples [55] showed the ability of PMA to eliminate free DNA or that from dead cells of biological samples.

Through the present study based on the metataxonomic analysis by Illumina 16S rRNA sequencing, we confirmed that the viable microbiome differs from the total bacterial profile in biofilm samples after exposure to an active antiseptic ingredient (Figure 2, Figure 3, Figure 4 and Figure 5). The study revealed that the PBS-treated samples as controls or with antiseptic but not exposed to the action of PMA showed no significant differences at any diversity level and thereby also showed no significant differences in the detected phylogenetic profiles (*p* > 0.05 in all cases), which impedes us from evaluating the effect of an antiseptic agent as a therapeutic intervention (Figure 2, Figure 3, Figure 4 and Figure 5). This result reaffirmed the inaccurate characterization of their composition and an overestimation of their richness in bacterial species or in their relative abundance [49,50,51,52]. Once the samples were exposed to PMA (the control samples and those treated with CHX/CPC), the efficacy of the combination of techniques was clearly demonstrated. We could then observe significant differences both at α- and β-diversity levels, coinciding with that described by other authors in various environments [36,49,50,51,52] including the oral cavity in saliva samples [55].

At the α-diversity level, we determined that, in agreement with that described in vivo, the cariogenic biofilms had a low α-diversity index with a very similar profile in ASVs [14]. When the biofilms were treated with a potent antiseptic and not exposed to PMA, the α-diversity profile showed no differences compared with the PBS-treated control (Figure 2). However, when applying the combination of massive sequencing and PMA to the samples treated with CHX/CPC, the effect of the antiseptic treatment revealed significant differences in the relative abundance in ASVs compared with the control (Figure 2).

When studying the β-diversity, we were able to confirm that, with the combination of Illumina 16S rRNA massive sequencing and PMA, the viable bacterial profile differed from the total profile in the oral biofilm samples after exposure to the antiseptic (Figure 3, Figure 4 and Figure 5). We confirmed that the sequencing technique by itself was unable to show what had occurred in the biofilms after exposure to the antiseptic treatment, returning a microbial profile with no significant differences compared with the PBS-treated control. Except for the samples belonging to the group treated with CHX/CPC and exposed to PMA, which differed considerably in their microbial composition from the rest (Figure 3a), the remaining samples showed increased homogeneity in their composition. Despite not clearly grouping according to the type of treatment applied, the samples belonging to the same group did appear to remain close to each other, as was the case for the samples treated with PBS unexposed to PMA and those exposed to PMA, as well as of the samples treated with CHX/CPC but not PMA (Figure 3). Therefore, the combination of massive sequencing and PMA allows us to verify that the bacterial community underwent significant changes in their β-diversity (Figure 2, Figure 3, Figure 4 and Figure 5).

Given that the study’s main objective was to determine whether massive sequencing techniques combined with PMA were able to assess the effect of a potent broad-spectrum antiseptic such as the combination of 0.12% CHX and 0.05% CPC, we were able to confirm their efficacy in the supragingival carious community. After the significant reduction in the viable bacterial load by more than three orders of magnitude (*p* = 0.048) (Table 1), which coincides with reports by other authors regarding the effectiveness of this antiseptic in dental plaque [6,57,58], we observed a considerable reduction in the phylum *Firmicutes* compared with the untreated biofilms (*p* > 0.05). A significant reduction was detected in the species *S. salivarius* (*p* < 0.001) and *S. parasanguinis* (*p* = 0.035) and an opposite trend for *Streptococcus* spp., which were significantly more abundant (*p* < 0.001). *S. salivarius* and *S. parasanguinis* have been related to the development of caries in the absence of *S. mutans* [12,13,14]. By reducing the abundance of the phylum *Firmicutes*, the presence of other phyla reached a relatively notable abundance, particularly the phylum *Actinobacteriota* after exposure to the antiseptic (*p* < 0.001), a phylum that includes genera such as *Actinomyces*, a quintessential early colonizer in dental plaque, and *Corynebacterium* [14]. Similarly, by reducing the presence of certain species of streptococci, other species acquired a more prominent role in terms of relative abundance, as was the case for *Corynebacterium*, *Staphylococcus*, *Prevotella*, and *Cutibacterium*, typical species in supragingival plaque (*p* < 0.05 in all cases) [14,59]. Recent studies have considered Corynebacterium a cornerstone in the development of supragingival plaque [60,61]. The evidence indicates *Corynebacterium* as a potential characteristic species in dental health [61,62,63], with a relevant role in plaque stability. Commensal microorganisms, such as certain streptococci and *Corynebacterium* spp., perform crucial roles in maintaining oral health through mechanisms that involve hydrogen peroxide production and the secretion of membrane vesicles, which can inhibit pathogenic species and modulate the host’s immune responses. Recent studies focused on the mechanisms of molecular commensalism have expanded our understanding of these key functions of the commensal microbiome, demonstrating its central role in oral health promotion and disease prevention [64]. Lastly, given the predominance of the genus *Streptococcus* and the involvement of various species in the development of oral cariogenic disease, we examined whether this was a single clone of the genus *Streptococcus* that was dominating the biofilm samples or whether there were several. We determined how the effect of the antiseptic reduced the total streptococcal load, reducing the viability of species related to the development of caries including *S. salivarius* and *S. parasanguinis*, considered as potentially responsible for dental caries disease in its various stages [9,10,11,12,13,14].

## 4. Materials and Methods

### 4.1. Development of the Ex Vivo Model of a Dysbiotic Oral Biofilm

The project was submitted for approval to the Ethics Committee of the San Carlos Clinical Hospital (C.I. 22/638-E). To implement the project, we employed a previously validated, static, ex vivo, cariogenic, supragingival biofilm model [59]. Briefly, the ex vivo biofilm model was performed on multiwell cell culture plates (Greiner Bio-one, Frickenhausen, Germany) from saliva collected orally and systemically from healthy volunteers [59]. A preliminary step consisted of pre-coating the wells with sterile saliva for 2 h at 36 ± 1 °C to promote the formation of the acquired film, following the descriptions by Sánchez et al. (2022) [59]. After this pre-incubation, we inoculated 20 μL of the pooled saliva from the saliva samples collected from the volunteers, also adding 2 mL of brain heart infusion medium (BHI; Becton, Dickinson and Company, Franklin Lakes, NJ, USA). For the caries-compatible dysbiotic biofilm condition, we supplemented it with 0.5% sucrose (Sigmoid, St. Louis, MO, USA). The plates were incubated without perturbation for 96 h at 36 ± 1 °C in aerobic conditions with 5.5% ultra-pure carbon dioxide (CO_2_) gasifying the atmosphere (Carbon dioxide Premier-X40S, Carburos, Air Products, Cornellá de Llobregat, Spain) to allow for the formation of biofilms [59].

The experiment was conducted on 3 separate occasions, with different saliva inocula, with 4 copies of the biofilm on each occasion (N = 12).

### 4.2. Exposure of the Biofilms to the Antiseptic

We employed the PerioAid mouthwash treatment (Dentaid, Cerdanyola, Spain), which contains 0.12% CHX and 0.05% CPC as active ingredients and has no alcohol in its formulation (CHX/CPC). PBS was employed as the positive control.

When the cariogenic biofilms were ready after 96 h of incubation, the supernatant was removed, and the wells were gently washed with PBS 3 times to eliminate the unattached bacteria (10 s per wash). Subsequently, half of the biofilms (N = 6) were exposed to 2 mL of the mouthwash, while the other half (N = 6) were exposed to 2 mL of PBS as the control, an exposure that lasted 60 s. After exposure, the mouthwash or PBS was removed by pipetting, and the wells were gently washed 3 consecutive times with 2 mL of PBS for 10 s to remove potential product residue. Subsequently, the biofilms were removed from the surface by successive and energetic pipetting with 1 mL of sterile PBS in the well and were transferred to a sterile plastic tube to then be disaggregated by vigorous vortex shaking for 3 min.

### 4.3. Exposure of Biofilms to Propidium Monoazide (PMA)

After the antiseptic treatment (or PBS as the control) and to discriminate between the DNA of the live and dead bacteria, we employed the PMA PMAxx™ (Biotium Inc., Fremont, CA, USA). From each of the 12 biofilms, 6 treated with CHX/CPC and 6 controls treated with PBS, we used 2 aliquots of 250 µL. The remaining 500 µL was centrifuged at 12,000 rpm for 3 min, the supernatant was removed, and the pellet was stored at −20 °C. Of the two aliquots, one did not undergo treatment with PMA and the other was exposed to this DNA intercalating compound, following a previously validated protocol for oral biofilms with certain modifications [30]. Briefly, PMA was added to a 250 µL aliquot of the disaggregated biofilms at a final concentration of 100 µM. The samples were then incubated for 10 min at 4 °C in the dark. The samples were then photoactivated for 30 min using the PMA-Lite LED Photolysis Device (Biotium Inc.), causing a cross-reaction between the DNA of the non-viable cells and the PMA. Another of the 250 µL aliquots of the disaggregated biofilms followed the same process but without the addition of PMA. The PMA-treated or untreated samples were then centrifuged at 12,000 rpm for 3 min, before proceeding with the DNA extraction.

### 4.4. Bacterial DNA Extraction of the Biofilms

The bacterial DNA was isolated from all samples using the MolYsis Complete5 commercial kit (Molzym Gmbh & Co. KG., Bremen, Germany) following the manufacturer’s instructions. The extracted DNA was diluted in 100 μL of sterile water (Roche Diagnostic GmbH, Mannheim, Germany) and stored at −20 °C until its subsequent analysis. A blank employed as a negative control, which did not include any sample, was subjected to all steps of the procedure described for DNA extraction.

The quality of the extracted DNA was verified by NanoDrop (NanoDrop One; ThermoFisher Scientific, Waltham, MA, USA) to then quantify it by the Qubit method (Qubit 2.0 Fluorometer; Invitrogen, Carlsbad, CA, USA).

### 4.5. qPCR Quantification of the Biofilm Bacteria

To quantify the bacteria included in the biofilms and subjected to the respective treatments, we employed the qPCR technique, with the use of 5′ nuclease hydrolysis probes. For the qPCR amplification, we employed universal primers and probes directed at gene 16S rRNA for total bacteria [30].

The reaction was conducted in a final volume of 10 µL, containing 5 µL of Master Mix 2x (LC 480 Probes Master; Roche, Basel, Switzerland), the ideal concentration of primers and probes (900, 900, and 300 nM, respectively, for total bacteria), and 2 µL of DNA of the biofilm samples. The negative control was 2 µL of sterile water (Water, PCR grade; Roche).

The amplification conditions consisted of an initial cycle of denaturation at 95 °C for 10 min, followed by 45 cycles at 95 °C for 15 s and 60 °C for 1 min. The analyses were performed with a QuantStudio Flex thermocycler (Applied Biosystems, Foster City, CA, USA). Every DNA sample was analyzed in duplicate. The quantification cycle (Cq) value was determined using QuantStudio Real-Time PCR Software (v1.3; Applied Biosystems).

The colony-forming units (CFU)/mL concentrations for each sample were determined by comparing the threshold value (Cq) with the Cq value of a standard curve of known bacterial concentrations, which were developed from 1 mL of saliva inoculum employed in the study, at a concentration of 109 CFU/mL. Within the acceptable PCR efficiency range of 80% to 120%, the reactions were designed and performed according to the minimum information for publication of quantitative real-time PCR experiments (MIQE) [65].

### 4.6. Preparation of the Illumina MiSeq Library

The composition of the microbial community was studied using the next-generation sequencing (NGS) technique, amplifying the hypervariable region V3–V4 of 16S gene rRNA from metagenomic DNA using the universal primers 8F (5′-AGAGTTTGATCCTGGCTCAG-3′) and 1492R (5′-CGGTTACCTTGTTACGACTT-3′). We employed the MiSeq 300 system (Illumina Inc., San Diego, CA, USA), with a ×2 focus. The PCR cycling conditions included initial denaturation at 95 °C for 3 min, followed by 25 cycles of amplification (30 s at 95 °C, 30 s at 55 °C, and 30 s at 72 °C). Amplification was performed using the KAPA HiFi HotStart ReadyMix PCR kit (KK2602) (Roche Diagnostics, Indianapolis, IN, USA), and a final extension step was conducted at 72 °C for 5 min, as described by Satari et al. (2020) [66]. Next, Illumina sequencing barcoded adaptors from the Nextera XT index kit v2 (FC-131-2001) (llumina Inc.) were combined with 16S rRNA amplicons. The resulting libraries were then normalized and merged. Finally, sequencing was conducted using paired-ends on an Illumina MiSeq platform (2 × 300 bp) at the Foundation for the Promotion of Health and Biomedical Research of the Valencian Community (Fisabio) (Valencia, Spain). A negative control of the DNA extraction was included, as well as a positive control of a simulated community to ensure quality control.

The analysis was performed in the Darwin Bioprospecting Excellence S.L. platform (Paterna, Valencia, Spain). Illumina sequencing data were processed using Qiime2 (v. 2022.11.0) [56] to conduct an initial quality control process on the sequences with DADA2. Sequence-quality assessments were performed using Qiime2 plugin demux (v. 2023.5.0). Trimming, joining, chimera removal, and amplicon sequence variant (ASV) detection (>99.9% similarity) were performed using the Qiime2-integrated DADA2 pipeline (v. 2023.5.0). The taxonomic assignment of each sequence variant was determined using the classify-Sklearn module of the feature classifier plugin (v. 2023.5.0) with SILVA (v. 138) as the reference database.

### 4.7. Statistical Data Analysis

The variables DNA concentration (ng/µL) and number of bacteria in the biofilms (CFU/mL) were subjected to the Shapiro–Wilk test to determine whether they resulted from a normal probability distribution. For the DNA concentration (ng/µL) when rejecting homoscedasticity using Levene’s test, we subsequently applied Welch’s and Brown–Forsythe’s robust analysis of variance, followed by Tamhane’s post hoc T2 test to determine the intergroup differences. For the number of bacteria, we applied Kruskal–Wallis’ non-parametric comparison for independent samples and Dunn’s post hoc test with Bonferroni correction to find significant intergroup differences. The analysis was performed using IBM SPSS Statistics v.29. Statistical significance was established at the 95% level of confidence with a *p*-value < 0.05.

The analysis of microbial ecology and statistical tests were performed using various R packages, including Phyloseq [67] and Vegan (https://CRAN.R-project.org/package=vegan, accessed on 11 March 2024) and visualized using ggplot2 (v. 3.4.0) and ampvis2 (v. 2.7.2). To minimize the possible bias resulting from the different sequencing depths among the samples, the abundance data were normalized with the total sum scaling approximation. The differential abundance analysis of the taxa was performed using the MaAsLin2 R package (v. 1.0.0) [68].

The Wilcoxon rank-sum test was used to test for significant differences at the alpha-diversity level. Beta-diversity analysis was conducted using principal component analysis (PCoA) based on Bray–Curtis dissimilarities to evaluate the similarity of the microbial communities. PERMANOVA tests were calculated using the adonis2 function from the vegan R package (v. 2.6.4) (https://CRAN.R-project.org/package=vegan, accessed on 11 March 2024) to detect statistically significant differences in the composition of the microbiome between the groups analyzed. The differential abundance analyses between taxa were conducted using the MaAsLin2 R package (v. 1.0.0) [68].

## 5. Conclusions

In conclusion and in agreement with reports from other studies, we have shown that PMA (employing culture-independent techniques) is an effective resource for solving the problem of differentiating viable cells from cells whose membranes are compromised or dead. PMA is a DNA intercalating compound that cannot be translocated through a viable cell membrane, which has enabled us to discriminate viable bacterial cells from non-viable ones, with high efficiency using the Illumina 16S rRNA sequencing technique. The results demonstrate the efficacy of PMA for selectively eliminating the DNA of dead prokaryotic cells during amplification of 16S gene rRNA in processes aimed at detecting and quantifying bacterial species in oral biofilms, as has been described for different environments.

## Figures and Tables

**Table 1:** Bacterial DNA concentrations, expressed as mean and standard deviation (SD) in ng/µL and quantified by Qubit, and the number of bacteria, expressed as mean and SD in CFU/mL and quantified by qPCR, from the biofilms treated for 60 s with phosphate buffered saline (PBS) (control biofilms) or with chlorhexidine/cetylpyridinium (CHX/CPC) antiseptic (treated biofilms) and then exposed or not to propidium monoazide (PMA) (final concentration 100 µM). Intergroup comparison performed with Tamhane’s T2 test for the DNA concentration and using Dunn’s test with Bonferroni correction for the bacterial count; a–c: different letters indicate a significant difference (*p* < 0.05 in both cases).

Biofilm Samples (N = 24)	Treatment (60 s)	Exposure to PMA (100 µM)	DNA Concentration (ng/µL) per Biofilm Mean (SD)	CFUs per Biofilm Mean (SD)
S_01 to S_012 (N = 12)	PBS	Untreated	14.1 (4.4) ^a^	1.4 × 10^9^ (6.1 × 10^8^) ^a^
		Treated	5.9 (2.4) ^a,b^	1.0 × 10^9^ (9.7 × 10^8^) ^b^
S_025 to S_036 (N = 12)	CHX/CPC	Untreated	3.1 (1.7) ^a,c^	9.0 × 10^8^ (6.4 × 10^8^) ^c^
		Treated	0.16 (0.1) ^a,b,c^	5.0 × 10^6^ (1.1 × 10^7^) ^a,b,c^

## Data Availability

The original contributions presented in the study are included in the article. Further inquiries can be directed to the corresponding author.
